# A Long Way to Travel

**Published:** 2005-09-15

**Authors:** Lynne S. Wilcox

The travel experiences of one Lemuel Gulliver were first published in 1726 (1). Captain Gulliver, an ordinary, middle-class Englishman, has a series of remarkable adventures, beginning with his trip to the island of Lilliput. Gulliver awakes on the island after surviving a shipwreck to discover that tiny human beings have tied him to the earth. Among the Lilliputians, he is kept shackled until he swears not to run away, to provide physical labor, and to fight the Lilliputians' enemies. These are the elements most people recognize as *Gulliver's Travels*, written by Jonathan Swift. Less often remembered are Gulliver's encounters with several more populations.

On his second trip, Gulliver is abandoned by his shipmates as they paddle for their lives at the sight of a Brobdingnagian, a giant as tall as a "spire-steeple." Gulliver meets a giant farmer who makes money exhibiting him at taverns, fairs, and inns. Gulliver eventually escapes back to England to embark on a third voyage. This time he finds himself on the flying island of Laputa. Here the men of the ruling class are so preoccupied with theoretical geometry that they are unaware of ordinary conversations. Gulliver has inferior status because he speaks to those who listen: women, tradesmen, and servants. He leaves Laputa and begins a fourth voyage. This time the good captain is forced into a longboat by mutineers and left in the land of the Houyhnhnms. These beings are sentient horses, while the humans on the island are Yahoos, "brute" animals without rational thought. The Houyhnhnms consider a Yahoo who is capable of communication to be a curiosity, but at length they conclude Gulliver is too dangerous and insist that he leave.

In all of these extraordinary cultures, it is Gulliver himself who is the disadvantaged one. Although he's treated in different ways depending on the values of each culture, he is an outsider to all. His experiences on the margins of these societies transform Gulliver, and he begins to see the norms of his own home in a new light. His final adventure with the Houyhnhnms changes his views on his own culture so profoundly that, upon his return home, he rejects the company of his fellow humans and, in a darkly satirical conclusion, prefers to spend his time with the horses in the stable. Ironically, his own culture perceives him as a madman, an outcast. Swift's cautionary tale demonstrates the destructive impact of cultural norms on health and well-being through Gulliver's frustration with and ultimate despair of finding a solution to the challenges presented in his own culture.

Although Gulliver's story does not report problems of health, it illustrates the challenge of disparity in select populations. For example, compared with white populations in the United States, African Americans have higher rates of breast cancer and cardiovascular deaths (2), American Indians have higher rates of diabetes (2), Hispanic populations have lower rates of mammography (3), and Asian Americans have lower rates of cervical Pap tests (4). Similar health disparities are reported in other cultures: in Mexico, the poorest populations have life expectancies 20 years shorter than those of the wealthiest; in Nigeria, life expectancy in the Borno region is 18 years less than in the Bendel region; in India, females under the age of 2 years are almost twice as likely to die as males; and ethnic differences in child mortality have been documented in many African countries (5).

The U.S. report *Healthy People 2010* identifies the elimination of racial and ethnic disparities in health as a major goal for 2010 (6). This issue of *Preventing Chronic Disease* provides commentaries and reports on the health of minority populations. Both Taylor et al (7) and Horn et al (8) examine health conditions among American Indians. This ethnic group is heterogeneous, with each tribe possessing its own language, culture, and health risks. This group's specialized needs are difficult to capture in national surveys because of small sample sizes, so it is essential to adopt methodology that collects valid data at the community level.

Taylor et al use community powwows for assessing health attitudes about diabetes among American Indians in Oklahoma, an important step in developing effective interventions. Horn et al use local schools in North Carolina to determine whether a national program to reduce smoking among teenagers can be successfully adapted for American Indian adolescents. Horn's pilot study raises questions, but it too is a first step in understanding American Indian health.

Latinos are one of the most rapidly growing populations in the United States (9). Welsh et al (10) use church congregations in Colorado to compare the success of personalized health education with simple distribution of printed materials to increase mammography rates among Latinas. These two programs varied little in results, suggesting that other interventions must be developed to improve low mammography rates among Hispanic women.

Ethnicity is one of many characteristics that creates health disparities. Johnson et al (11) describe a collaborative intervention in rural areas of Montana and Wyoming. Public health departments provided technical expertise to local physicians in screening and prevention management, and the physicians were able to significantly improve several diabetes control and immunization rates among their patients. The project illustrates the potential for public health and medical care partnerships to overcome geographic disadvantage.

U.S. public health does not have to retreat in despair to the horse stables as Gulliver did. The findings from these reports can aid us in recognizing problems early and sustaining effective responses. Unlike Gulliver, we can work to change cultural norms destructive to health and well-being. But we still have a long way to travel. Public health leaders must be wary of simple solutions and understand that removing one layer of health disparities may reveal others. As Kumanyika points out in her thoughtful essay on African American women and obesity (12), multiple conditions can affect the same people and communities. These concerns require hard questions and holistic responses.
